# 5-Benzyl-7-methyl­hexa­hydro-3a,7-methano-1*H*-furo[3,4-*c*]azocine-3,10(4*H*)-dione

**DOI:** 10.1107/S160053681100300X

**Published:** 2011-02-05

**Authors:** Zhan-Kun Yang, Feng-Peng Wang

**Affiliations:** a Department of Chemistry of Medicinal Natural Products, West China College of Pharmacy, Sichuan University, Chengdu 610041, People’s Republic of China

## Abstract

The title compound, C_18_H_21_NO_3_, was obtained *via* a double Mannich condensation reaction of 6-methyl­tetra­hydro­isobenzofuran-1,7(3*H*,7a*H*)-dione with formaldehyde and benzyl­amine. The mol­ecule contains three fused rings of which the cyclo­hexa­none and piperidine rings adopt chair conformations and the furan­one ring assumes an envelope conformation. An inter­molecular C—H⋯π inter­action is present in the crystal structure.

## Related literature

For the double Mannich condensation reaction, see: Guthmann *et al.* (2009 ▶); Coates *et al.* (1994 ▶); Barker *et al.* (2002 ▶). For the methyl­ation of the β-keto ester in the synthesis of the title compound, see: Weiler (1970 ▶).
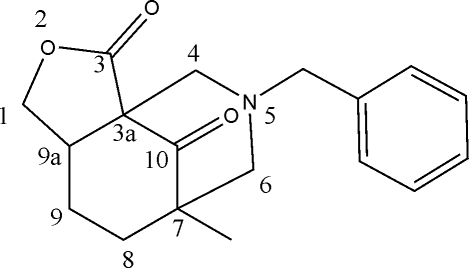


## Experimental

### 

#### Crystal data

C_18_H_21_NO_3_
*M* *_r_* = 299.36
Orthorhombic, 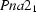

*a* = 10.795 (2) Å
*b* = 14.386 (3) Å
*c* = 9.797 (2) Å
*V* = 1521.5 (5) Å^3^
*Z* = 4
Mo *K*α radiation
μ = 0.09 mm^−1^
*T* = 293 K
0.20 × 0.20 × 0.20 mm

#### Data collection

Rigaku Saturn 724 diffractometer
10268 measured reflections
1584 independent reflections
1546 reflections with *I* > 2σ(*I*)
*R*_int_ = 0.045

#### Refinement

*R*[*F* ^2^ > 2σ(*F* ^2^)] = 0.067
*wR*(*F* ^2^) = 0.167
*S* = 1.16
1584 reflections
200 parameters
1 restraint
H-atom parameters constrained
Δρ_max_ = 0.15 e Å^−3^
Δρ_min_ = −0.16 e Å^−3^

### 

Data collection: *CrystalClear* (Rigaku/MSC, 2005 ▶); cell refinement: *CrystalClear*; data reduction: *CrystalClear*; program(s) used to solve structure: *SHELXS97* (Sheldrick, 2008 ▶); program(s) used to refine structure: *SHELXL97* (Sheldrick, 2008 ▶); molecular graphics: *ORTEP-3 for Windows* (Farrugia, 1997 ▶); software used to prepare material for publication: *SHELXL97*.

## Supplementary Material

Crystal structure: contains datablocks I, global. DOI: 10.1107/S160053681100300X/xu5143sup1.cif

Structure factors: contains datablocks I. DOI: 10.1107/S160053681100300X/xu5143Isup2.hkl

Additional supplementary materials:  crystallographic information; 3D view; checkCIF report

## Figures and Tables

**Table 1:** Hydrogen-bond geometry (Å, °) *Cg* is the centroid of the phenyl ring.

*D*—H⋯*A*	*D*—H	H⋯*A*	*D*⋯*A*	*D*—H⋯*A*
C8—H8*B*⋯*Cg*^i^	0.97	2.87	3.833 (6)	169

Symmetry code: (i) 
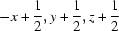
.

**Fig. 1.**
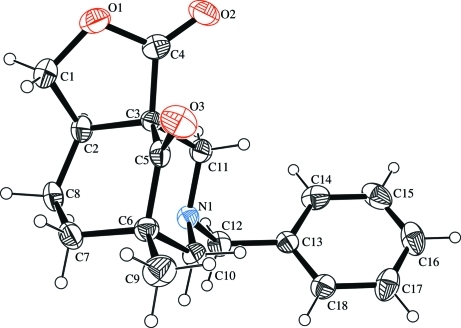
The molecular structure of the title compound, showing the atom-numbering scheme with displacement ellipsoids at 30% probability level.

## supplementary crystallographic information

### Comment

The AE rings of diterpenoid alkaloids have received much attention as key intermediate in the total syntheses of diterpenoid alkaloids. Double Mannich condensation (Guthmann *et al.*, 2009; Coates *et al.*, 1994; Barker *et al.*, 2002) is an efficient method to append the E ring to the A ring. Therefore, we have designed and synthesized the racemic 1-substituted AE-bicyclic analogue by double Mannich condensation. Herein, we report the structure of the title compound.

As illustrated in Fig. 1, the molecule of the title compound is constructed from the fusion of a cyclohexanone ring, a piperidine ring and a furanone ring. The two six-membered rings are in standard chair conformations. The furanone ring is *cis*-fused with the cyclohexanone ring and adopts envelope conformation. The bond angles around C4 and C5 are indicative of *sp*^2^ hybridization for the two atoms. And the strain in the furanone ring is illustrated by the much distorted triangular geometry of C4 atom and the bond angles around C4 range between 109.7 (4) and 128.6 (5)°.

### Experimental

The intermediate, 6-methyltetrahydroisobenzofuran-1,7(3H,7aH)-dione (1b), was synthesized according to the procedure described by Weiler (1970). A solution of tetrahydroisobenzofuran-1,7(3H,7aH)-dione (1.00 g, 6.49 mmol) in THF (10 mL) was added to 1M lithium diisopropylamide solution in THF (14.2 ml, 14.2 mmol) at 273 K. After 30 min, CH_3_I (0.48 ml, 7.71 mmol) was added dropwise in the mixture. Then the mixture was stirred at the same temperature for 2 h. H_2_O (20 mL) was added and the solution was extracted with CH_2_Cl_2_ (60 mL). The organic layer was dried over anhydrous Na_2_SO_4_ and concentrated under reduced pressure. The crude product was purified by flash column chromatography (ethyl acetate/hexane, v:v, 1:2) to give 1b. (0.382 g, yield 35%) as a colourless oil.

To a solution of 1b (200 mg, 1.19 mmol) in EtOH (300 mL) was added 37% CH_2_O solution (0.29 mL, 3.57 mmol) and phenylmethanamine (195 µL, 1.79 mmol). The reaction mixture was refluxing for 48 h and then concentrated under reduced pressure. The crude product was purified by flash column chromatography (ethyl acetate/hexane, v:v, 1:4) to give the title compound (107 mg, yield 30%) as a white solid. Crystallization from a ethyl acetate-petroleum ether system yielded colourless crystals suitable for single-crystal structure determination.

### Refinement

H atoms were fixed geometrically and treated as riding, with C—H = 0.98 (methine), 0.97 (methylene), 0.96 (methyl) or 0.93 Å (aromatic) and *U*_iso_(H) = 1.5*U*_eq_(C) for methyl groups and *U*_iso_(H) = 1.2*U*_eq_(C) for the others. A total of 1163 Friedel pairs were merged before final refinement as there is no significant anomalous dispersion for the determination of the absolute configuration.

### Crystal data

**Table:**

C_18_H_21_NO_3_	*F*(000) = 640
*M**_r_* = 299.36	*D*_x_ = 1.307 Mg m^−^^3^
Orthorhombic, *P**n**a*2_1_	Mo *K*α radiation, λ = 0.71073 Å
Hall symbol: P 2c -2n	Cell parameters from 3565 reflections
*a* = 10.795 (2) Å	θ = 2.5–27.5°
*b* = 14.386 (3) Å	µ = 0.09 mm^−^^1^
*c* = 9.797 (2) Å	*T* = 293 K
*V* = 1521.5 (5) Å^3^	Prism, colourless
*Z* = 4	0.20 × 0.20 × 0.20 mm

### Data collection

**Table:**

Rigaku Saturn 724 diffractometer	1546 reflections with *I* > 2σ(*I*)
Radiation source: fine-focus sealed tube	*R*_int_ = 0.045
graphite	θ_max_ = 26.0°, θ_min_ = 2.5°
ω scans	*h* = −12→13
10268 measured reflections	*k* = −17→17
1584 independent reflections	*l* = −10→12

### Refinement

**Table:**

Refinement on *F*^2^	Secondary atom site location: difference Fourier map
Least-squares matrix: full	Hydrogen site location: inferred from neighbouring sites
*R*[*F*^2^ > 2σ(*F*^2^)] = 0.067	H-atom parameters constrained
*wR*(*F*^2^) = 0.167	*w* = 1/[σ^2^(*F*_o_^2^) + (0.0752*P*)^2^ + 0.8271*P*] where *P* = (*F*_o_^2^ + 2*F*_c_^2^)/3
*S* = 1.16	(Δ/σ)_max_ < 0.001
1584 reflections	Δρ_max_ = 0.15 e Å^−^^3^
200 parameters	Δρ_min_ = −0.16 e Å^−^^3^
1 restraint	Absolute structure: unk
Primary atom site location: structure-invariant direct methods	

### Special details

**Table:**

Experimental. For 6-methyltetrahydroisobenzofuran-1,7(3H, 7aH)-dione (1b), ^1^H NMR (400 MHz, CDCl_3_): δ 4.28 (dd, J = 9.2, 4.8 Hz, 1H), 4.15 (d, J = 9.2 Hz, 1H), 3.46(d, J = 7.2 Hz,1H), 2.97–2.91 (m, 1H), 2.40–2.34 (m, 1H), 2.07–2.03 (m, 2H), 1.79–1.69 (m, 1H), 1.49–1.40 (m, 1H), 1.09(d, J = 6.0 Hz, 3H); ^13^C NMR (100 MHz CDCl_3_): δ 204.3, 172.2, 72.1, 54.4, 44.0, 40.7, 32.5, 26.9, 14.2.For 5-benzyl-7-methylhexahydro-1H-3a,7-methanofuro [3,4-c]azocine- 3,10(4H)-dione (1), ^1^H NMR (400 MHz, CDCl_3_): δ 7.37–7.27(m, 5H), 4.29 (t, J = 9.2 Hz, 1H), 3.83 (dd, J =9.2, 10.4 Hz, 1H), 3.61, 3.51 (ABq, J = 13.0 Hz, 2H), 3.14–3.12(m, 1H), 3.07, 2.85 (ABq, J = 11.2 Hz, 2H), 3.05, 2.38 (ABx, J = 2.4, 12.0 Hz, 2H), 2.81–2.75 (m, 1H), 2.26–2.20 (m, 1H), 1.92–1.87 (m, 1H), 1.44–1.38 (m, 1H), 0.99 (s, 3H); ^13^C NMR (100 MHz, CDC_l3_): δ 210.7, 173.4, 137.7, 128.7, 128.5, 127.5, 69.2, 65.8, 61.5, 59.8, 58.6, 47.5, 46.1, 39.2, 22.0, 20.7
Geometry. All e.s.d.'s (except the e.s.d. in the dihedral angle between two l.s. planes) are estimated using the full covariance matrix. The cell e.s.d.'s are taken into account individually in the estimation of e.s.d.'s in distances, angles and torsion angles; correlations between e.s.d.'s in cell parameters are only used when they are defined by crystal symmetry. An approximate (isotropic) treatment of cell e.s.d.'s is used for estimating e.s.d.'s involving l.s. planes.
Refinement. Refinement of *F*^2^ against ALL reflections. The weighted *R*-factor *wR* and goodness of fit *S* are based on *F*^2^, conventional *R*-factors *R* are based on *F*, with *F* set to zero for negative *F*^2^. The threshold expression of *F*^2^ > σ(*F*^2^) is used only for calculating *R*-factors(gt) *etc*. and is not relevant to the choice of reflections for refinement. *R*-factors based on *F*^2^ are statistically about twice as large as those based on *F*, and *R*- factors based on ALL data will be even larger.

### Fractional atomic coordinates and isotropic or equivalent isotropic displacement parameters (Å^2^)

**Table:**

	*x*	*y*	*z*	*U*_iso_*/*U*_eq_	
O1	0.4992 (4)	1.0732 (2)	0.3736 (5)	0.0579 (10)	
O2	0.6479 (3)	0.9870 (3)	0.2802 (5)	0.0630 (11)	
O3	0.5495 (4)	0.8809 (3)	0.5463 (4)	0.0635 (11)	
N1	0.3480 (3)	0.7803 (3)	0.2454 (4)	0.0389 (9)	
C1	0.3691 (5)	1.0624 (4)	0.4104 (7)	0.0556 (14)	
H1B	0.3216	1.1164	0.3825	0.067*	
H1A	0.3601	1.0542	0.5082	0.067*	
C2	0.3259 (4)	0.9760 (3)	0.3343 (5)	0.0424 (11)	
H2	0.3083	0.9938	0.2398	0.051*	
C3	0.4465 (4)	0.9178 (3)	0.3349 (5)	0.0346 (10)	
C4	0.5441 (5)	0.9930 (4)	0.3236 (5)	0.0443 (11)	
C5	0.4612 (4)	0.8689 (3)	0.4713 (5)	0.0371 (11)	
C6	0.3570 (4)	0.8020 (3)	0.4992 (5)	0.0405 (11)	
C7	0.2386 (4)	0.8628 (4)	0.5110 (6)	0.0483 (12)	
H7A	0.1679	0.8219	0.5225	0.058*	
H7B	0.2452	0.9004	0.5929	0.058*	
C8	0.2129 (4)	0.9273 (4)	0.3903 (6)	0.0465 (12)	
H8A	0.1754	0.8912	0.3176	0.056*	
H8B	0.1532	0.9739	0.4186	0.056*	
C9	0.3758 (6)	0.7484 (4)	0.6313 (6)	0.0606 (15)	
H9B	0.4532	0.7157	0.6276	0.091*	
H9A	0.3094	0.7046	0.6428	0.091*	
H9C	0.3765	0.7909	0.7068	0.091*	
C10	0.3545 (5)	0.7331 (3)	0.3766 (6)	0.0442 (11)	
H10B	0.4286	0.6950	0.3792	0.053*	
H10A	0.2835	0.6923	0.3858	0.053*	
C11	0.4512 (4)	0.8433 (3)	0.2246 (5)	0.0401 (11)	
H11B	0.4455	0.8718	0.1350	0.048*	
H11A	0.5289	0.8096	0.2300	0.048*	
C12	0.3250 (5)	0.7190 (4)	0.1294 (6)	0.0476 (12)	
H12B	0.3046	0.7575	0.0513	0.057*	
H12A	0.2523	0.6819	0.1500	0.057*	
C13	0.4275 (4)	0.6538 (3)	0.0878 (5)	0.0386 (11)	
C14	0.5080 (6)	0.6763 (4)	−0.0179 (6)	0.0583 (15)	
H14	0.4984	0.7322	−0.0644	0.070*	
C15	0.6027 (6)	0.6160 (5)	−0.0545 (7)	0.0690 (19)	
H15	0.6555	0.6315	−0.1260	0.083*	
C16	0.6189 (6)	0.5334 (5)	0.0141 (7)	0.0663 (18)	
H16	0.6829	0.4934	−0.0098	0.080*	
C17	0.5400 (6)	0.5112 (4)	0.1175 (7)	0.0635 (17)	
H17	0.5504	0.4554	0.1641	0.076*	
C18	0.4448 (5)	0.5703 (3)	0.1542 (6)	0.0470 (12)	
H18	0.3916	0.5535	0.2246	0.056*	

### Atomic displacement parameters (Å^2^)

**Table:**

	*U*^11^	*U*^22^	*U*^33^	*U*^12^	*U*^13^	*U*^23^
O1	0.071 (2)	0.0449 (19)	0.058 (2)	−0.0122 (18)	0.002 (2)	−0.005 (2)
O2	0.045 (2)	0.072 (3)	0.072 (3)	−0.0190 (19)	0.010 (2)	−0.007 (2)
O3	0.052 (2)	0.083 (3)	0.056 (3)	−0.010 (2)	−0.0164 (19)	0.003 (2)
N1	0.038 (2)	0.040 (2)	0.039 (2)	0.0011 (17)	−0.0009 (17)	−0.0094 (17)
C1	0.059 (3)	0.043 (3)	0.065 (4)	0.010 (2)	0.010 (3)	−0.006 (3)
C2	0.041 (2)	0.045 (3)	0.042 (3)	0.011 (2)	−0.004 (2)	−0.001 (2)
C3	0.032 (2)	0.035 (2)	0.037 (2)	−0.0028 (18)	0.0027 (18)	−0.0030 (18)
C4	0.053 (3)	0.044 (3)	0.037 (3)	−0.007 (2)	−0.007 (2)	−0.005 (2)
C5	0.031 (2)	0.043 (2)	0.038 (3)	0.0065 (19)	−0.0019 (19)	−0.009 (2)
C6	0.044 (2)	0.038 (2)	0.040 (3)	−0.002 (2)	0.002 (2)	0.001 (2)
C7	0.038 (2)	0.055 (3)	0.052 (3)	0.000 (2)	0.010 (2)	−0.009 (3)
C8	0.034 (2)	0.056 (3)	0.050 (3)	0.015 (2)	0.002 (2)	−0.005 (3)
C9	0.077 (4)	0.059 (3)	0.045 (3)	0.002 (3)	0.003 (3)	0.007 (3)
C10	0.044 (2)	0.037 (2)	0.052 (3)	−0.004 (2)	0.002 (2)	−0.012 (2)
C11	0.042 (2)	0.038 (2)	0.040 (3)	0.000 (2)	0.000 (2)	−0.005 (2)
C12	0.040 (2)	0.053 (3)	0.050 (3)	−0.001 (2)	−0.007 (2)	−0.013 (2)
C13	0.037 (2)	0.040 (2)	0.039 (3)	−0.009 (2)	−0.006 (2)	−0.013 (2)
C14	0.075 (4)	0.055 (3)	0.045 (3)	−0.012 (3)	0.010 (3)	−0.011 (3)
C15	0.058 (3)	0.084 (5)	0.065 (4)	−0.018 (3)	0.021 (3)	−0.033 (4)
C16	0.052 (3)	0.078 (4)	0.068 (4)	0.012 (3)	−0.004 (3)	−0.041 (4)
C17	0.069 (4)	0.051 (3)	0.070 (4)	0.014 (3)	−0.022 (4)	−0.022 (3)
C18	0.055 (3)	0.042 (3)	0.044 (3)	−0.004 (2)	−0.001 (2)	−0.010 (2)

### Geometric parameters (Å, °)

**Table:**

O1—C1	1.458 (7)	C8—H8A	0.9700
O1—C4	1.344 (6)	C8—H8B	0.9700
O2—C4	1.202 (6)	C9—H9B	0.9600
O3—C5	1.216 (6)	C9—H9A	0.9600
N1—C10	1.455 (7)	C9—H9C	0.9600
N1—C11	1.451 (6)	C10—H10B	0.9700
N1—C12	1.460 (6)	C10—H10A	0.9700
C1—H1B	0.9700	C11—H11B	0.9700
C1—H1A	0.9700	C11—H11A	0.9700
C1—C2	1.523 (7)	C12—H12B	0.9700
C2—H2	0.9800	C12—H12A	0.9700
C2—C3	1.548 (6)	C12—C13	1.507 (7)
C2—C8	1.509 (7)	C13—C14	1.389 (8)
C3—C4	1.513 (7)	C13—C18	1.380 (7)
C3—C5	1.519 (7)	C14—H14	0.9300
C3—C11	1.523 (6)	C14—C15	1.388 (9)
C5—C6	1.505 (7)	C15—H15	0.9300
C6—C7	1.552 (7)	C15—C16	1.376 (10)
C6—C9	1.520 (8)	C16—H16	0.9300
C6—C10	1.558 (7)	C16—C17	1.362 (10)
C7—H7A	0.9700	C17—H17	0.9300
C7—H7B	0.9700	C17—C18	1.382 (8)
C7—C8	1.529 (8)	C18—H18	0.9300
			
O1—C1—H1B	110.7	C6—C10—H10A	109.1
O1—C1—H1A	110.7	C7—C6—C10	113.7 (4)
O1—C1—C2	105.2 (4)	C7—C8—H8A	108.6
O1—C4—C3	109.7 (4)	C7—C8—H8B	108.6
O2—C4—O1	121.8 (5)	H7A—C7—H7B	107.4
O2—C4—C3	128.6 (5)	C8—C2—C1	116.7 (4)
O3—C5—C3	123.2 (4)	C8—C2—H2	107.9
O3—C5—C6	124.5 (5)	C8—C2—C3	115.3 (4)
N1—C10—C6	112.7 (4)	C8—C7—C6	115.7 (4)
N1—C10—H10B	109.1	C8—C7—H7A	108.4
N1—C10—H10A	109.1	C8—C7—H7B	108.4
N1—C11—C3	108.4 (4)	H8A—C8—H8B	107.6
N1—C11—H11B	110.0	C9—C6—C7	109.4 (4)
N1—C11—H11A	110.0	C9—C6—C10	109.6 (4)
N1—C12—H12B	107.9	H9B—C9—H9A	109.5
N1—C12—H12A	107.9	H9B—C9—H9C	109.5
N1—C12—C13	117.5 (4)	H9A—C9—H9C	109.5
C1—C2—H2	107.9	C10—N1—C12	114.5 (4)
C1—C2—C3	100.4 (4)	H10B—C10—H10A	107.8
H1B—C1—H1A	108.8	C11—N1—C10	112.2 (4)
C2—C1—H1B	110.7	C11—N1—C12	113.5 (4)
C2—C1—H1A	110.7	C11—C3—C2	113.9 (4)
C2—C8—C7	114.6 (4)	H11B—C11—H11A	108.4
C2—C8—H8A	108.6	H12B—C12—H12A	107.2
C2—C8—H8B	108.6	C13—C12—H12B	107.9
C3—C2—H2	107.9	C13—C12—H12A	107.9
C3—C11—H11B	110.0	C13—C14—H14	119.8
C3—C11—H11A	110.0	C13—C18—C17	120.9 (6)
C4—O1—C1	110.2 (4)	C13—C18—H18	119.5
C4—C3—C2	101.5 (4)	C14—C13—C12	121.1 (5)
C4—C3—C5	108.9 (4)	C14—C15—H15	119.8
C4—C3—C11	115.3 (4)	C15—C14—C13	120.5 (6)
C5—C3—C2	109.9 (4)	C15—C14—H14	119.8
C5—C3—C11	107.1 (4)	C15—C16—H16	120.4
C5—C6—C7	105.6 (4)	C16—C15—C14	120.5 (6)
C5—C6—C9	112.3 (4)	C16—C15—H15	119.8
C5—C6—C10	106.2 (4)	C16—C17—H17	119.5
C6—C5—C3	112.2 (4)	C16—C17—C18	120.9 (7)
C6—C7—H7A	108.4	C17—C16—C15	119.1 (6)
C6—C7—H7B	108.4	C17—C16—H16	120.4
C6—C9—H9B	109.5	C17—C18—H18	119.5
C6—C9—H9A	109.5	C18—C13—C12	120.9 (5)
C6—C9—H9C	109.5	C18—C13—C14	118.0 (5)
C6—C10—H10B	109.1	C18—C17—H17	119.5
			
O1—C1—C2—C3	32.6 (5)	C5—C6—C7—C8	54.1 (6)
O1—C1—C2—C8	158.1 (4)	C5—C6—C10—N1	−53.3 (5)
O3—C5—C6—C7	117.6 (5)	C6—C7—C8—C2	−41.8 (6)
O3—C5—C6—C9	−1.6 (7)	C7—C6—C10—N1	62.4 (5)
O3—C5—C6—C10	−121.3 (5)	C8—C2—C3—C4	−160.5 (4)
N1—C12—C13—C14	−97.1 (6)	C8—C2—C3—C5	−45.4 (5)
N1—C12—C13—C18	82.4 (6)	C8—C2—C3—C11	74.9 (6)
C1—O1—C4—O2	176.2 (5)	C9—C6—C7—C8	175.2 (5)
C1—O1—C4—C3	−4.9 (6)	C9—C6—C10—N1	−174.8 (4)
C1—C2—C3—C4	−34.2 (5)	C10—N1—C11—C3	−61.7 (5)
C1—C2—C3—C5	80.9 (5)	C10—N1—C12—C13	−69.9 (6)
C1—C2—C3—C11	−158.8 (4)	C10—C6—C7—C8	−62.0 (6)
C1—C2—C8—C7	−81.1 (6)	C11—N1—C10—C6	58.3 (5)
C2—C3—C4—O1	25.5 (5)	C11—N1—C12—C13	60.8 (6)
C2—C3—C4—O2	−155.7 (6)	C11—C3—C4—O1	149.2 (4)
C2—C3—C5—O3	−120.4 (5)	C11—C3—C4—O2	−32.0 (8)
C2—C3—C5—C6	61.7 (5)	C11—C3—C5—O3	115.3 (5)
C2—C3—C11—N1	−59.9 (5)	C11—C3—C5—C6	−62.6 (4)
C3—C2—C8—C7	36.4 (6)	C12—N1—C10—C6	−170.3 (4)
C3—C5—C6—C7	−64.6 (5)	C12—N1—C11—C3	166.5 (4)
C3—C5—C6—C9	176.2 (4)	C12—C13—C14—C15	179.5 (5)
C3—C5—C6—C10	56.5 (5)	C12—C13—C18—C17	−179.0 (5)
C4—O1—C1—C2	−18.5 (6)	C13—C14—C15—C16	−0.6 (9)
C4—C3—C5—O3	−10.1 (6)	C14—C13—C18—C17	0.6 (7)
C4—C3—C5—C6	172.1 (4)	C14—C15—C16—C17	0.7 (9)
C4—C3—C11—N1	−176.7 (4)	C15—C16—C17—C18	−0.2 (9)
C5—C3—C4—O1	−90.4 (5)	C16—C17—C18—C13	−0.5 (8)
C5—C3—C4—O2	88.4 (6)	C18—C13—C14—C15	0.0 (7)
C5—C3—C11—N1	61.9 (5)		

### Hydrogen-bond geometry (Å, °)

**Table:**

Cg is the centroid of the phenyl ring.

**Table:**

*D*—H···*A*	*D*—H	H···*A*	*D*···*A*	*D*—H···*A*
C8—H8B···Cg^i^	0.97	2.87	3.833 (6)	169

Symmetry codes: (i) −*x*+1/2, *y*+1/2, *z*+1/2.

## References

[bb1] Barker, D., Brimble, M. A., Mcleod, M., Savage, G. P. & Wong, D. J. (2002). *J. Chem. Soc.* **7**, 924–931.

[bb2] Coates, P. A., Blagbrough, I. S., Rowan, M. G., Potter, B. V. L., Pearson, D. P. J. & Lewis, T. (1994). *Tetrahedron Lett.* **35**, 8709–8712.

[bb3] Farrugia, L. J. (1997). *J. Appl. Cryst.* **30**, 565.

[bb4] Guthmann, H., Conol, D., Wright, E., Koerber, K., Barker, D. & Brimble, M. A. (2009). *Eur. J. Org. Chem.* **12**, 1944–1960.

[bb5] Rigaku/MSC (2005). *CrystalClear* Rigaku/MSC, The Woodlands, Texas, USA.

[bb6] Sheldrick, G. M. (2008). *Acta Cryst.* A**64**, 112–122. 10.1107/S0108767307043930 18156677

[bb7] Weiler, L. (1970). *J. Am. Chem. Soc.* A**92**, 6702–6704.

